# Diagnostic Utility of a Modified Reticulin Algorithm in Pediatric Adrenocortical Neoplasms

**DOI:** 10.1097/PAS.0000000000002174

**Published:** 2023-12-29

**Authors:** Oscar Lopez-Nunez, Calogero Virgone, Irina S. Kletskaya, Luisa Santoro, Stefano Giuliani, Bruce Okoye, Marco Volante, Andrea Ferrari, Gianni Bisogno, Eleonora Duregon, Mauro Papotti, Gianluca De Salvo, Sarangarajan Ranganathan, Rita Alaggio

**Affiliations:** * Department of Pathology and Laboratory Medicine, University of Pittsburgh Medical Center, Pittsburgh, PA; ‡ Department of Women’s and Children's Health, University of Padua; ¶ Pathology Unit, Department of Medicine DIMED; §§ Department of Women’s and Children’s Health, University of Padua; § Pediatric Surgery, University Hospital of Padua; ∥∥ Pediatric Hematology-Oncology Division, University Hospital of Padua, Padua; ## Clinical Research Unit, Istituto Oncologico Veneto IOV IRCCS, Padua; †† Department of Oncology, Pediatric Oncology Unit, University of Turin, San Luigi Hospital, Orbassano; ¶¶ Division of Pathology, Department of Oncology, University of Turin, at “Città. della Salute e della Scienza” Hospital, Turin; ‡‡ Pediatric Oncology Unit, Fondazione IRCCS Istituto Nazionale dei Tumori, Milan, Italy; *** Pathology Unit, Department of Laboratories, Bambino Gesù Children’s Hospital, IRCCS; ††† Department of Medical-Surgical Sciences and Biotechnologies, Sapienza University of Rome, Polo Pontino, Rome, Italy; ∥ Russian Children’s Clinical Hospital of Pirogov, Russian National Research Medical University, Moscow, Russia; # Department of Specialist Neonatal and Pediatric Surgery, Great Ormond Street Hospital for Children NHS Foundation Trust; ** Department of Pediatric Surgery, St George’s Hospital London, London; † Department of Pediatrics, University of Cincinnati College of Medicine, Cincinnati, OH; Division of Pathology and Laboratory Medicine, Cincinnati Children’s Hospital Medical Center, Cincinnati, OH; Department of Pathology and Laboratory Medicine, University of Cincinnati College of Medicine, Cincinnati, OH

**Keywords:** adrenocortical tumors, pediatric, Wieneke score, carcinoma, reticulin, algorithm, prognosis

## Abstract

Pediatric adrenocortical neoplasms (ACNs) are extremely rare tumors in contrast to their adult counterparts. Distinguishing benign from malignant is challenging based on pure morphologic grounds. Previously, 2 scoring systems were proposed in pediatric ACN, including the Wieneke criteria (WC) and its modified version (modified WC [mWC]). In adults, the reticulin algorithm (RA) has proven inexpensive, reliable, predictive, and reproducible; however, it has been validated only recently in children in a limited number of cases. This study aims to assess the RA utility compared with other scoring systems in a series of 92 pediatric ACNs. All cases were individually scored, and mitotic rate cutoffs were recorded. Reticulin alterations were classified as quantitative and qualitative. Outcome data were available in 59/92. The median age was 5 years (0.1 to 18 y) with an M:F of 0.6. Clinical presentation included virilization (39%), Cushing syndrome (21%), other symptoms (4%), and asymptomatic (36%). The reticulin framework was intact in 27% and altered in 73% of cases, showing qualitative (22%), quantitative (73%), and both (5%) alterations. In patients with favorable outcomes, 59% showed either intact reticulin or qualitative alteration compared with the unfavorable outcome group, where 90% showed quantitative alterations. All scoring systems WC (*P* < 0.0001), mWC (*P* = 0.0003), and the adult/pediatric RA (*P* < 0.0001) had predictive value. The RA is comparable to WC and mWC, easier to apply, and is the most sensitive histopathological approach to identifying aggressive behavior in pediatric ACN. Its integration into the WC might be helpful in ACN of uncertain malignant potential and deserves further investigation.

Adrenocortical neoplasms (ACNs) are rare tumors in children, representing only 0.2% of all pediatric malignancies and 5 to 6% of all adrenal tumors.^1^ Among pediatric populations, ACN tends to display a biphasic age distribution, with an initial peak observed within the first decade of life (infantile group) and a second peak observed in adolescents.^2–4^ Interestingly, a female predominance is noted in both groups while differing in clinical presentation and prognosis.^5^ Besides age at presentation, other pathologic and clinical features have been consistently recognized as prognostic markers in pediatric ACN, including tumor size and weight,^2,3,5–11^ Ki67 proliferative index,^1,12^ and clinical stage.^1,9,10,13^ Conversely, the prognostic role of pure histopathologic assessment remains elusive. As a matter of fact, despite their frequent atypical morphology, most pediatric ACNs show indolent behavior (in contrast to their adult counterparts), making the morphologic distinction between benign and malignant lesions particularly challenging, as no single histopathologic parameter appears to be predictive of malignancy and outcome.^2,3^ Consequently, the role of histopathology has been relegated as the main guidance for treatment. To address this issue, Wieneke et al^3^ proposed a scoring system based on 9 histomorphological features in a series of 83 pediatric ACNs. This scoring system was progressively recognized as the Wieneke criteria (WC) and appears to be predictive of clinical behavior in pediatric ACN, having been validated in other series.^9,10,12,14,15^ More recently, Picard et al^1^ revisited the role of WC in assessing pediatric ACN, emphasizing that some of these criteria remain poorly defined among pathologists and questioning its prognostic specificity. This group proposed a modified version of the WC (modified WC [mWC] or 5-item microscopic score), defined by a new 2-step scoring algorithm (including Ki67 proliferative index) demonstrating prognostic and therapeutic implications,^16^ yet requiring independent validation.

A common denominator among most scoring systems in ACN, regardless of age, is that they are multiparametric, time-consuming, and often challenging to apply in clinical practice, prompting the validation of more straightforward approaches. In 2009, some of us^17^ proposed the reticulin algorithm (RA) as a simplified method in a series of adult ACNs demonstrating a similar performance to the more conventional Weiss criteria but faster and easier to implement. This method comprises a stepwise approach requiring initial confirmation of a reticulin framework disruption by conventional histochemical stain, followed by identification of at least 1 out of 3 malignancy criteria (necrosis, mitotic rate >50/50 high-power fields [HPFs] and/or venous invasion), ultimately leading to a diagnosis of malignancy (ie, adrenocortical carcinoma). The RA was further validated by Duregon et al^18^ in a multicentric study, confirming its high sensitivity, specificity, interobserver reproducibility, and ability to predict outcomes. Other series have reported equivalent findings;^19–24^ however, pediatric cases were not sufficiently represented. This study aims to validate the RA diagnostic utility, as compared with the WC and recently proposed mWC, in a series of pediatric ACNs.

## PATIENTS AND METHODS

### Study Cohort

Institutional and consultation formalin-fixed paraffin-embedded tissue blocks from 92 pediatric ACN specimens were queried from the surgical pathology files at 5 large academic hospitals after Institutional Review Board approval. Only specimens from patients aged 0 to 18 years old containing sufficient available clinical information and histopathological material were selected for the current series. Patients were classified according to the Children’s Oncology Group staging system, and, before starting the study, all cases were anonymized and only coded data were used throughout.^17^

### Microscopic, Immunohistochemical, and Digital Analysis

Hematoxylin-eosin stained sections and available immunohistochemical material were reviewed. All tumors were confirmed to be adrenal cortical in origin based on morphologic features and immunoreactivity for alpha inhibin, Melan A, HMB45, and synaptophysin immunostains. In addition, one representative section per case was selected to perform reticulin histochemical stain (Artisan Reticulin/Nuclear Fast Red Stain Kit, AR#179; Silver Stain kit, Bio Optica) and Ki67 immunohistochemistry (mouse monoclonal antibody MIB1, 1:100 dilution, DAKO). All specimens were scanned at ×40 magnification using the Leica Aperio AT2 scanner (Leica Microsystems Inc.), and virtual extension files (1 hematoxylin-eosin, 1 Ki67-immunohistochemical, and 1 reticulin stain per case) were uploaded to the Histoscan server for central review. All cases were independently and blindly scored by at least 2 pediatric pathologists according to the original WC (benign vs uncertain vs malignant),^3^ mWC (5-item microscopic score, favorable vs unfavorable histology),^1^ and RA (benign vs malignant).^17,18^ In addition, the RA was subclassified based on the corresponding mitotic count per HPFs. The latter was recorded by using the adult cutoff as per the Weiss criteria (>5/50 HPF; further designated as adult RA [aRA]),^25,26^ as well as the pediatric cutoff as per the WC (>15/20 HPF; further designated as pediatric RA [pRA]).^3^

The reticulin stain was performed at each participating institution using a commercially available silver impregnation-based kit. The sections were examined under both low and high magnification. A section of the normal adrenal gland was selected as a positive control (intact reticular framework) and defined as a uniform fibrillary arrangement completely surrounding adrenal cortical cells in nests and cords. Disruptions of the reticular framework were scored as previously described.^17,18^ Briefly, alterations were classified as quantitative (loss of fibers in extensive areas) and qualitative (apparently intact framework with frayed fibers of irregular thickness surrounding groups of neoplastic cells, or fibers surrounding individual cells).

The Ki67 proliferative index, as one of the key parameters of the mWC, was digitally evaluated by selecting regions of interest within the tumor area by one of the authors. Necrotic and nonviable cell areas were excluded from the analysis. The proliferative index assessment was performed based on the Aperio nuclear V9 (Aperio Technologies) algorithm for nuclear immunoreactivity. In any given slide, the total number of positive nuclei was divided by the total number to obtain the proliferative index with a cutoff of ≥15%, as previously described.^1^ In selected cases, manual verification was carried out by identification of hot spot areas containing positive neoplastic cells at ×40 magnification. Digital images from these areas were captured and printed to conduct a manual count of positive nuclei on at least 2000 tumor cells per case. The proliferative index was reported as the percentage of positive nuclei.

### Statistical Analyses

All data were analyzed using descriptive statistical methods. Fifty-nine patients had available follow-up information, and an outcome analysis was conducted. Statistical analysis included Cohen *K*, Cramer *V* coefficient, Kaplan-Meier survival curves with log-rank test, Cox regression, and logistic regression.

Kaplan-Meier curves were built, and a log-rank test was used to assess the association between each score and the outcome. A *P* value <0.05 was considered statistically significant. Cramer *V* and Cohen *K* were calculated to assess the agreement between the different score systems in this study. Cohen *K* coefficient was calculated to assess the levels of agreement between pRA, aRA, and mWC. *K* values were evaluated as follows: 0 to 0.20, no agreement; 0.21 to 0.39, minimal agreement; 0.40 to 0.59, weak agreement; 0.6 to 0.79, moderate agreement; 0.80 to 0.90, strong agreement; 0.91 to 1.00, almost perfect agreement. Cramer *V* was calculated to determine the measure of association between Wieneke score and pRA, aRA, and mWC. *V* values between 0 and 0.3 indicated a weak association, between 0.3 and 0.6 a good association, and between 0.6 and 1 a strong association. The risk proportional hazard model (Cox model), with survival time as the dependent variable, and logistic regression with evaluation of areas under the receiver operating characteristic curves, were used to compare the relative value of the scores, according to the Akaike Information Criterion (AIC). The model with the lowest AIC value was considered to be the “best” model.

All statistical analyses were performed with SAS version 9.4 (SAS Institute Inc.).

## RESULTS

### Clinical Findings

In total, 92 ACNs were obtained from institutional and consultation files. The cohort distribution per age group was 49% (<5 y), 19% (≥5 to <10 y), and 32% (≥10 y) with a median age of 5 years (range: 0.1 to 18 y). The male-to-female ratio was 0.6. The clinical presentation included virilization (39%), Cushing syndrome (21%), other symptoms (4%), and palpable mass or incidental diagnosis (36%). There were syndromic (Li-Fraumeni syndrome, Beckwith-Wiedemann syndrome, and Prader-Willi syndrome) or other associated medical conditions (anorectal malformation, unspecified nephropathy, unspecified immunodeficiency, human immunodeficiency virus–acquired immunodeficiency syndrome, and Asperger syndrome) in 15% of patients. The outcome information (present patient status) was available for review in 59 cases. The median follow-up was 48 months with the following distribution: complete remission (n = 39), alive with disease (n = 9), and died of disease (n=11).

### Pathologic Findings

**FIGURE 1:**
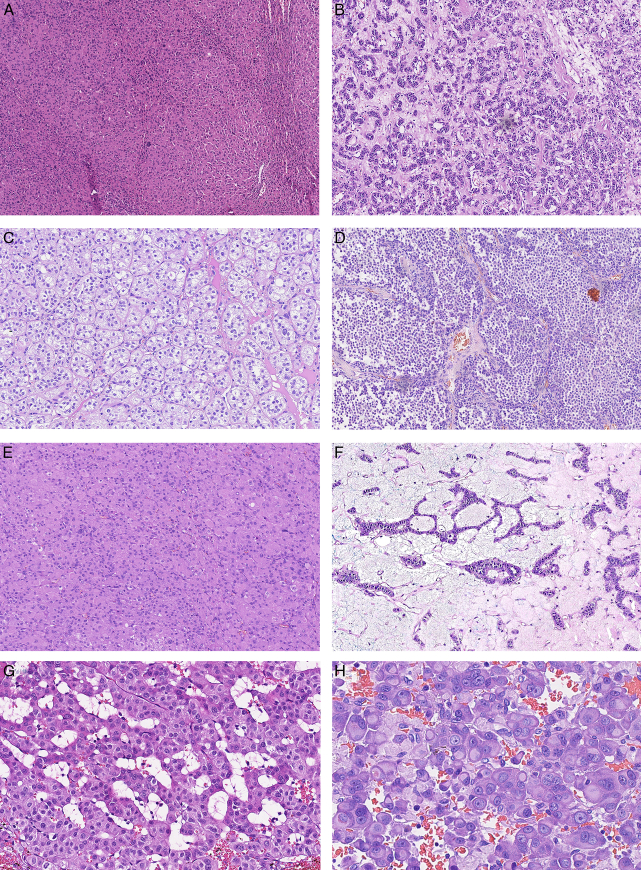
Predominant morphologic features of 92 ACNs, including diffuse/solid (A), trabecular (B), nested (C), and alveolar (D) patterns. A subset of tumors also had prominent oncocytic features (E) and variable myxoid stroma (F). Myelolipomatous metaplasia (G) and signet-ring-cell-like cytomorphology (H) were also occasionally present (hematoxylin-eosin stain).

The average tumor size was 7.04 cm (0.5 to 25 cm), whereas the average tumor weight was 228.1 g (6 to 3750 g). The majority of tumors arose from the left adrenal gland (56%). The predominant histomorphologic patterns included diffuse/solid (40%, 37/92), trabecular (32%, 29/92), nested (26%, 24/92), and alveolar (2%, 2/92). A subset of tumors also had prominent oncocytic features (23%, 21/92) and variable myxoid stroma (9%, 8/92). One tumor showed extensive myelolipomatous metaplasia, whereas another exhibited signet-ring-cell-like cytomorphology (Fig. 1). Additional morphologic features are reported in Table 1.

**TABLE 1:** Pathologic Features of 92 Pediatric ACNs

Features	Value	Missing data	%
Macroscopic findings			
Location			
Right adrenal	37	7	44
Left adrenal	48	—	56
Weight (g)			
Median (minimum—maximum)	60 (6–3750)	47	—
Size (cm)			
Median (minimum–maximum)	6 (0.5–25)	7	—
Microscopic findings			
Vascular invasion	28	—	—
Capsular invasion	17	5	—
High mitotic count (>5/50 HPF)	55	4	63
High mitotic count (>15/20 HPF)	22	—	—
Atypical mitoses	31	5	—
Necrosis	43	—	—
Ki67 >15%	15	15	—
Wieneke score (WC)	—	1	—
<3 benign	52	—	57
3 indeterminate	17	—	19
>3 malignant	22	—	24
Modified Wieneke score (mWC)	—	32	—
Favorable histology	46	—	—
Unfavorable histology	14	—	—
aRA	—	7	—
Benign	39	—	—
Malignant	46	—	—
pRA	—	7	—
Benign	47	—	—
Malignant	38	—	—

**FIGURE 2:**
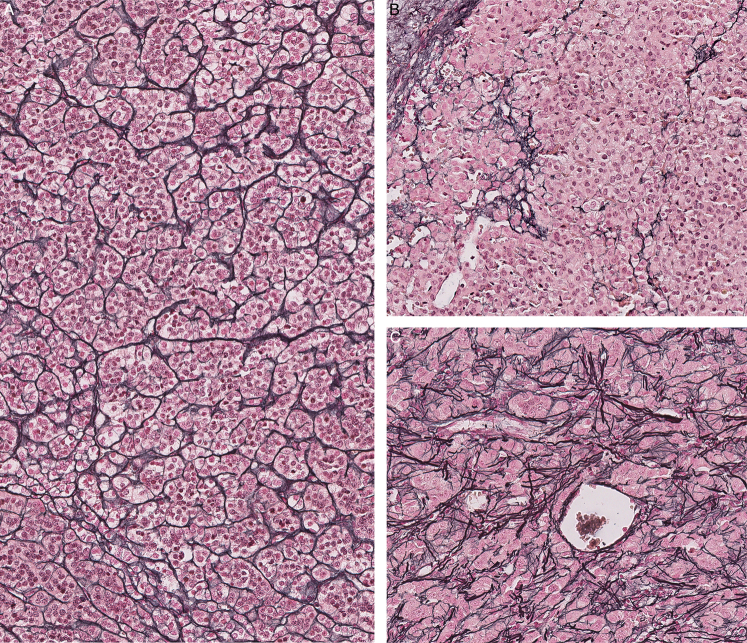
Histochemical analysis of the reticulin framework in adrenocortical tumors demonstrating examples of the intact framework (A), compared with disruption with quantitative (B) and qualitative (C) alterations (reticulin stain).

**FIGURE 3:**
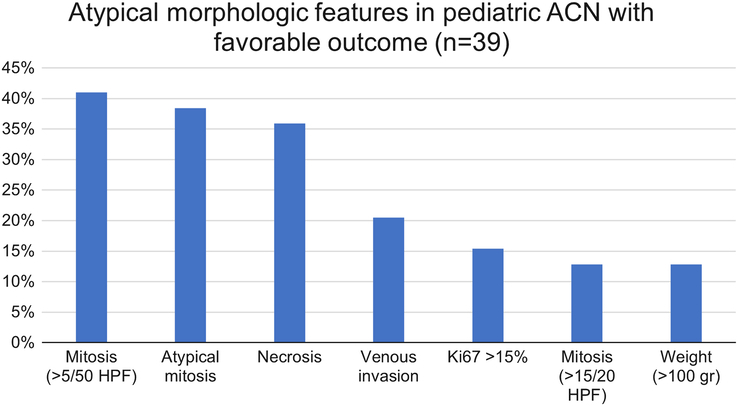
Spectrum and frequencies of altered morphologic features identified in several pediatric ACNs with otherwise favorable outcomes.

The reticulin framework was defined as intact (27%, 25/92 cases) and altered (73%, 67/92 cases). Among cases with reticulin alteration, the distribution was qualitative (22%, 15/67 cases), quantitative (73%, 49/67 cases), and both (5%, 3/67 cases; Fig. 2). The distribution per clinical stage was 50% (stage I), 29.5% (stage II), 9% (stage III), and 11.5% (stage IV) among tumors with available outcome information. Most patients with favorable outcomes had either intact or qualitative-only reticulin alterations (59%). In contrast, most patients with unfavorable outcomes had documented quantitative reticular alterations (90%). Several ACNs with favorable outcomes showed a spectrum of atypical morphologic features (ie, brisk mitosis, atypical mitosis, necrosis, etc; Fig. 3). Two tumors with predominant myxoid morphology and available follow-up had unfavorable outcomes (alive with disease and died of disease).

### Histopathologic Scoring Systems

All scoring systems (WC, mWC, aRA, and pRA) were applied and interpreted among pediatric patients with available outcome information (n = 59). The distribution of cases according to WC was benign (53%), uncertain malignant potential (15%), and malignant (32%). According to the mWC, cases were classified as favorable histology (76%) and unfavorable histology (24%). The distribution of cases was deemed as benign (42%) and malignant (58%) according to the aRA whereas benign (49%) and malignant (51%) according to the pRA. Additional clinicopathological features according to different scoring systems are summarized in Table 2 and Table 3.

**TABLE 2:** Histological Features According to Different Scoring Algorithms in Patients With Available Follow-up Data (n = 59)

	mWC		aRA		pRA	
	Favorable	Unfavorable	Benign	Malignant	Benign	Malignant
Reticulin framework status						
Intact	11	0	11	0	11	0
Quantitative alteration	20	12	4	28	5	27
Qualitative alteration	12	2	10	4	13	1
Both	2	0	0	2	0	2
Microscopic criteria						
>5 mitosis/50 HPF	24	11	6	29	10	25
>15 mitosis/20 HPF	8	7	1	14	1	14
Necrosis	19	13	2	30	4	28
Vascular invasion	7	11	3	15	4	14
Capsular invasion	6	5	2	9	3	8
Ki67 >15%	4	5	2	7	2	7
Wieneke score						
Benign	30	1	23	8	25	6
Undetermined	7	2	1	8	3	6
Malignant	8	11	1	18	1	18

**TABLE 3:** Clinical Features According to Outcome in Patients With Available Follow-up Data (n = 59)

	mWC		aRA		pRA	
	Favorable	Unfavorable	Benign	Malignant	Benign	Malignant
Sex						
Male	17	6	9	14	10	13
Female	28	8	16	20	19	17
Median age (mo)	43	109	57.5	43.5	41	60
Laterality						
Left	24	7	13	18	17	14
Right	18	6	11	13	11	13
NA	3	1	1	3	1	3
Median size (cm)	5.5	11.8	5	8	5	9.3
Median weight (g)	51.5	90	31.5	76.5	40	73
Stage						
I	29	1	18	12	20	10
II	11	4	4	11	5	10
III	4	3	2	5	3	4
IV	0	6	0	6	0	6
NA	1	0	1	0	1	0
Outcome						
CR	35	4	24	15	28	11
AWD	5	4	1	8	1	8
DOD	5	6	0	11	0	11

AWD indicates alive with disease; CR, complete remission; DOD, died of disease; NA, not available.

All scoring systems were predictive of prognosis (WC, *P* < 0.001; mWC, *P* = 0.0003; RA, *P* < 0.001) and associated with survival as shown by the Kaplan-Meier curves (log-rank test, *P* <0.0001 for all 4 scores). The Cohen *K* coefficient showed a strong agreement between pRA and aRA (0.86; 95% CI: 0.74-0.99) and a minimal agreement among the mWC, pRA and aRA (0.39; 95% CI: 0.20-0.59 and 0.31; 95% CI: 0.13-0.49, respectively). Cramer *V* showed that WC has a strong agreement with aRA (0.68) and pRA (0.69) and a good agreement with the mWC (0.57). Areas under the receiver operating characteristic curve, obtained with logistic regression, were estimated to be 0.8534 for the WC, 0.8446 for the pRA, 0.7920 for aRA, and 0.6855 for the mWC.

The AIC values according to the Cox model and logistic regression were estimated, with the pRA score showing the lowest AIC value in both methods (Table 4).

**TABLE 4:** AIC Values for Each Prognostic Score

Prognostic score	Cox model	Logistic regression
pRA	120.018	50.891
WC	120.461	55.097
aRA	126.032	58.467
mWC	134.044	70.805

aRA indicates adult Reticulin algorithm; mWC, modified Wieneke criteria or 5-items microscopic score; pRA, pediatric Reticulin algorithm; WC, Wieneke criteria.

## DISCUSSION

Pediatric ACNs are rare tumors, which may pose important diagnostic and treatment challenges. In adults, the histologic assessment of ACN is typically based on one of several scoring systems/algorithms, such as the Weiss system or the Helsinki score.^27^ Children have a significantly better prognosis than their adult counterparts despite atypical histomorphology. Furthermore, it is well known that adult-based systems tend to overestimate the rate of malignancies in children.^2^ In the last 20 years, pediatric pathologists have relied on the WC.^28^ Although WC has been independently validated in various studies, a series recently reported relatively low accuracy for classifying pediatric ACN.^29^ Although a modified score (mWC) has been proposed, this system has been conceived only for a subset of pediatric ACN patients (stage II and III tumors) and requires independent validation.^1^ A common denominator among the available diagnostic scoring systems, regardless of age, is that they are time-consuming and often challenging to apply in clinical practice. Furthermore, the risk stratification into prognostic groups to drive treatment decisions is challenging in children with localized malignant ACN.^30^ Interestingly, the diagnostic and prognostic utility of the RA has been demonstrated in adults as a relatively simpler approach,^17,18^ and it was recently adopted for a small subset (28 cases) of pediatric patients.^31^ The authors reported a 100% sensitivity of the RA and that none of the other evaluated scores (WC, Linn-Weiss-Bisceglia criteria, and Ki67 score) could predict an unfavorable outcome with 100% accuracy; in addition, they adopted the RA as it was originally described.^17,18^ On these premises, the adoption of a pRA could be a first step towards a more precise diagnosis and stratification in this population, possibly paving the way to a better understanding of the existing relationship between histomorphology and clinical course.

In this study, we identified a slightly higher incidence of ACN in females, in concordance with previous pediatric series^30^ and we demonstrated the utility of the RA in the histologic assessment of pediatric ACN and confirmed its reproducibility and easier implementation among pathologists. Contrary to what was reported in adult ACN,^17,18^ we consistently identified reticulin alterations in tumors that were ultimately (and correctly) classified as benign according to both RA and pRA scoring systems. Along these lines, most qualitative-only reticulin alterations were seen in tumors of patients with a favorable outcome, in contrast to adult ACN, where all reported qualitative alterations were identified in malignant neoplasms.^18^ This discrepancy between age groups is not entirely clear; however, it could be related to divergent biological mechanisms in pediatric ACN in comparison to adult counterparts. Previously, others have described similar differences in matrix metalloproteinase 2 and HLA-class II expression in pediatric versus adult ACN;^17,32^ a notion that also reinforces the differences between age groups (molecular alterations, clinical manifestations, and prognosis) and the need for more stringent criteria in pediatric ACN.^33^

We have verified that atypical histomorphology is common in children with otherwise indolent courses and favorable outcomes and should not be used as a standalone diagnostic parameter in pediatric ACN. Others have hypothesized that pediatric ACNs are linked histogenetically to the developing adrenal gland as they arise from the fetal cortex (rather than from mature adult cortical cells) often leading to spontaneous regression/maturation and a benign course despite the presence of atypical histologic features.^2,29^ Among our patients with favorable outcomes, we found that the most common histologic alterations were high mitotic rate (>5/50 HPF), atypical mitoses, and necrosis. By using the reticulin-based scoring systems (aRA and pRA), abnormal features were present in both adrenocortical adenomas and carcinomas, although at different rates. A careful review of different histologic variants revealed only 2 cases with predominant myxoid morphology and available follow-up information in the present study. Interestingly, both patients had unfavorable outcomes, in correlation with previously reported findings by Magro et al,^32^ where malignant pediatric ACNs were frequently found to have myxoid stromal changes.

Both reticulin-based scoring systems were associated with survival in our pediatric patients, similar to the WC and mWC; the latter 2 being already validated elsewhere.^1,3,9,10,12,14,15^ Interestingly, when separately assessing WC and mWC against the reticulin-based scoring systems, we found a stronger correlation of WC with aRA/pRa compared with that of mWC with the reticulin scores. The difference between the aRA and the pRA is essentially related to the reference mitotic rate cutoff (the pRA was based on the cutoff points previously proposed and validated in the WC for pediatric ACN). Among these, the pRA was ultimately estimated to be the best model according to the AIC analysis. Our findings demonstrated that all implemented scoring systems were fairly predictive of outcomes in this series. Nonetheless, the pRA is clearly advantageous in this setting, as it is tailored to pediatric patients, reproducible, inexpensive, and easier to implement, as it has been shown already in adults.^17,18^ Further studies should be aimed at independent, prospective validation of the pRA in other series of pediatric ACNs, and the potential utility of its integration with traditional scoring systems. In addition, the pathologic stratification of pediatric ACN is an important step in treatment and follow-up decisions. This is particularly relevant in low-stage tumors that have been completely resected to assess the need and extent of adjuvant medical therapy. Although further risk stratification according to the tumor’s stage was not performed in the present study, this should be prospectively validated in further pediatric series, as it may ideally drive further therapeutic decisions, and allow modulating the intensity of chemotherapy treatment (eg, mitotane alone vs conventional chemotherapy plus mitotane in stage II patients).^30^

## CONCLUSIONS

The pRA is fast, reproducible, inexpensive, and easier to implement while assessing pediatric ACN, as demonstrated already in adult cohorts. Our data shows that the pRA shows at least equal predictive value when compared with other more complex pediatric scoring systems (ie, WC and mWC), with the non-negligible advantage to simplify the diagnostic process and its use should be encouraged in a prospective approach.
